# Mpox research coordination to effectively support response efforts in Africa: Lessons learned, challenges, and way forward

**DOI:** 10.4102/jphia.v17i1.1867

**Published:** 2026-07-31

**Authors:** Mosoka P. Fallah, Olayinka S. Ilesanmi, Abigael A. Mesfin, Shahd Osman, Oluwatoyosi Olawande, Tamrat Shaweno, Yap Boum, Nebiyu Dereje, Ngashi Ngongo, Jean Kaseya

**Affiliations:** 1 Department of Science and Innovation, Africa Centres for Disease Control and Prevention, Addis Ababa, Ethiopia; 2 Western Africa Regional Coordination Centre, Africa Centres for Disease Control and Prevention, Addis Ababa, Ethiopia; 3 School of Medicine, College of Health Sciences, Addis Ababa University, Addis Ababa, Ethiopia; 4 Southern Africa Regional Coordination Centre, Africa Centres for Disease Control and Prevention, Addis Ababa, Ethiopia; 5 Incident Management Support Team, Africa Centres for Disease Control and Prevention, Addis Ababa, Ethiopia; 6 Executive Office, Africa Centres for Disease Control and Prevention, Addis Ababa, Ethiopia

**Keywords:** mpox, Africa CDC, Research and Innovation, Africa, IMST, outbreak, evidence-based

## Abstract

Mpox, caused by the mpox virus, has evolved from isolated cases in endemic regions to widespread outbreaks affecting multiple countries in Africa. The Research and Innovation pillar of the Continental Incident Management Support Team (IMST) was established to generate evidence that supports response efforts, including analysing the efficiency and effectiveness of clinical trials and health research on mpox across Africa. This article describes the achievements, challenges faced and lessons learned from mpox research efforts. The contributions of research to the mpox response by the Africa Centres for Disease Control and Prevention (Africa CDC) are crucial to managing infectious disease outbreaks across the continent. Multiple research initiatives were coordinated across Member States, including socio-behavioural, epidemiological, diagnostic, vaccine and therapeutic studies. Key achievements included the establishment of a coordinated continental research platform, initiation of multi-country studies and mapping of ongoing mpox research activities across Africa. Challenges included bureaucratic procedures, delays in funding disbursement, ethical approval bottlenecks, coordination complexities and surveillance gaps that affected timely research implementation. Addressing these gaps has the potential to enhance evidence-based decision-making and provide valuable lessons for improving future epidemic preparedness and response in Africa.

**Contribution:**

The mpox research demonstrated the vitality of collaborative research efforts, the need for continuous, systematic monitoring of its progress and the need for real-time dissemination of research findings to inform practices that ensure an effective response to mpox. Real-time dissemination of research findings, starting with early community engagement, is critical to ensure that research findings are translated into action.

## Introduction

### Background

In response to the surge in mpox cases in 2024, the Africa Centres for Disease Control and Prevention (Africa CDC) declared a Public Health Emergency of Continental Security (PHECS) for the first time, following extensive consultations with key stakeholders, including the Advisory Technical Council and the Governing Board.^1,2^ Additional concerns included the need for better coordination of research activities, vaccine complexities, and limited diagnostic capacities. Following Africa CDC’s declaration, the World Health Organization (WHO) convened the International Health Regulations Emergency Committee on August 14, 2024, which unanimously declared the mpox upsurge a Public Health Emergency of International Concern (PHEIC).^1,3^ This declaration underscores the critical need for strengthened global collaboration and intensified response efforts to control the epidemic. The dominance of Clades Ib and Ia continued to fuel transmission, complicating containment efforts, particularly in regions with high cross-border mobility. Despite progress in some areas, gaps in surveillance, resource allocation and regional coordination existed.

Coordinating mpox research across Africa has been essential to addressing knowledge gaps and improving response efforts. A centralised platform serves to align research with global standards, fostering collaboration among African institutions. Rapid-response research projects explore knowledge, attitudes and practices (KAP), vaccine and therapeutic uptake and diagnostic improvements. Multi-country clinical trials assessed the effectiveness and safety of vaccines and treatments, particularly for vulnerable populations. This article describes the achievements, challenges faced and lessons learned from mpox research efforts.

## Research and Innovation pillar supporting response to mpox

Ten strategic response pillars, including leadership and coordination, surveillance, vaccination, laboratory, research, risk communication and community engagement (RCCE), continuity of essential healthcare services, case management, infection prevention and control (IPC) and logistics and supply chain, comprised the first Mpox Continental Response Plan created by the Incident Management Support Team (IMST).^4^ The Research and Innovation pillar of the Continental IMST was established to generate evidence that supports the response efforts, including analysing the efficiency and effectiveness of clinical trials and health research on mpox across Africa.^5^ This mechanism came out of the need for cohesiveness in managing mpox in Africa, where fragmented efforts often hinder progress. By addressing critical challenges such as stakeholder collaboration, resource mobilisation and the harmonisation of research standards, the mechanism seeks to strengthen Africa’s public health infrastructure and improve health outcomes across the continent. Notably, the establishment of the joint mechanism is significant for its potential to revolutionise mpox health research in Africa, particularly in the wake of the coronavirus disease 2019 (COVID-19) pandemic, which highlighted existing gaps in data collection, response coordination and clinical trial capabilities.

The Research and Innovation pillar of the IMST emphasised collaborative governance and aimed to streamline processes for research while fostering partnerships among government entities, civil society and international organisations. Furthermore, it has been serving as a centralised data-sharing platform to enhance transparency and communication among stakeholders involved in health research. Key functions of the pillar include the management and coordination of clinical trial activities, policy development to support health ministries and the mobilisation of resources to ensure effective research execution.

## Mpox research key achievements and best practices

Joint partners’ coordination meetings provided an opportunity to map and track all research to foster collaboration across disciplines for holistic insights. Africa CDC, in collaboration with World Health Organization (WHO), National Institutes of Health (NIH), and Coalition for Epidemic Preparedness Innovation (CEPI), together with many African scientists and ethicists, held a scientific conference on the alignment of research responses with outbreak goals. The conference, held on 29 August 2024 and 30 August 2024, with over 2500 participants, mostly Africans, came up with the following research priorities: The development of an emergency mpox research response, enhanced collaboration and partnerships.^6^ Mpox socio-behavioural studies were initiated in 10 countries. This multi-country involvement in research was a boost to the research being conducted at each member state level. Senior researchers from various institutions were able to contribute to the planning of the research and its execution. Examples of such studies include the mpox socio-behavioural and epidemiological study in Demographic Republic of the Congo (DRC), the epidemiological and clinical characteristics of mpox study in Burundi, and compassionate treatment with tecovirimat in progress in the Central African Republic. In addition, training and technical support were provided to country research teams on protocol implementation, research coordination, ethical compliance and data collection procedures prior to study initiation.

The research pillar of the IMST also maps and tracks research to foster collaboration across disciplines for holistic insights. This facilitated the integration of the diverse research foci, which were digitised and made open access. As of December 2024, a total of 48 studies on the continent had been recorded as shown in Table 1.^7^

**TABLE 1:** Mpox studies ongoing in Africa.

Study type	Number of studies	Key focus areas	Geographic scope
Epidemiology studies (including modelling, serosurveillance, genomics, outbreak investigations)	35	Transmission dynamics, clinical characterisation, seroprevalence, genomics, modelling, wastewater surveillance, risk factors, One Health investigations	Predominantly DRC; multi-country across Central, West, East and Southern Africa
Vaccine trials	6	Safety, immunogenicity, effectiveness, dose optimisation, post- and pre-exposure vaccination	DRC, Uganda, Nigeria, CAR, multi-country
Therapeutic trials	4	Clinical efficacy and safety of tecovirimat, brincidofovir, platform, and expanded-access protocols	DRC, CAR, Nigeria, multi-country
Diagnostic trials	2	Decentralised molecular diagnostics, validation of point-of-care tests	DRC, Nigeria
Socio-behavioural studies	1	Knowledge, attitudes, practices, socio-ecological and behavioural drivers of mpox transmission	Multi-country (Central, West, East, and Southern Africa)

*Source:* CEPI. Mpox outbreak and study sites [homepage on the Internet]. [cited 2026 Jan 09]. Available from: https://cepi-shiny-apps.shinyapps.io/mpox-map/

DRC, Demographic Republic of the Congo; CAR, Central African Republic.

Following the August 2024 research prioritisation conference, the Research pillar coordinated a series of activities throughout the response period. These included regular partner coordination meetings to monitor research progress, mapping and tracking of ongoing mpox studies across Africa, initiation of multi-country socio-behavioural studies in 10 countries and support for epidemiological, clinical, therapeutic and vaccine-related research initiatives. These activities facilitated collaboration among researchers, improved visibility of ongoing studies and informed evidence-based response efforts.

**FIGURE 1:**
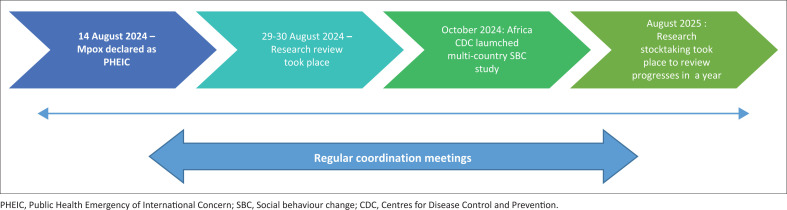
Timeline of activities undertaken by the research pillar of the mpox incident management support team.

A year later, in August 2025, Africa CDC, in collaboration with partners, conducted a stocktaking meeting on mpox research to assess progress made and challenges faced, as shown in Figure 1. By harmonising data collection across Member States, the Africa CDC coordinated regional public health initiatives, ensuring that comprehensive information on cases is shared promptly to enable effective resource mobilisation and real-time monitoring. Ultimately, these research contributions are integral to building resilient health systems capable of managing current and future public health challenges across the continent, highlighting the importance of international cooperation and collaboration in addressing the mpox crisis.

The response to the mpox outbreaks in Africa involves extensive partnerships and collaborations among various stakeholders, emphasising a multi-faceted and coordinated approach to public health emergencies.^5^ This collaborative effort is co-led by the Africa CDC and the WHO, along with other key partners such as United Nations (UN) agencies, non-governmental organisations and civil society groups. The WHO, in conjunction with the Africa CDC, is engaging a broad array of international, regional and national partners to strengthen coordination in areas of preparedness, readiness and response to mpox.^8^

Furthermore, Funding plays a crucial role in supporting these collaborative efforts. Africa CDC has been advocating for increased domestic investment and innovative financing to ensure sustained support for health initiatives, despite external funding challenges. A strong data-sharing framework will facilitate timely research dissemination, guiding public health strategies and policies. Local production of medical countermeasures will reduce dependence on imports, enhancing Africa’s capacity to combat mpox effectively.

## Challenges faced by the Research and Innovation pillar

The response to the mpox outbreak in Africa has been significantly hampered by a multitude of challenges, particularly in regions experiencing instability and limited resources. Countries such as the DRC face concurrent humanitarian crises and health emergencies that complicate focused responses to mpox, making it difficult to prioritise efforts amid community distrust and the need for effective risk communication strategies.

Delays in fund transfers to Member States have severely hampered the timely commencement of mpox research. Without prompt financial disbursement, essential activities such as fieldwork and personnel recruitment are stalled, leading to missed research timelines and weakened outbreak response. Bureaucratic procedures, stringent financial regulations and coordination challenges between donors and recipient countries further exacerbate the issue.^9^ To address this, establishing a more efficient fund disbursement mechanism, ensuring transparency in financial processes and enhancing coordination between Africa CDC and national institutions are critical for accelerating research implementation.

Delays in ethical approval from multiple ethics boards across different countries have significantly hindered the timely launch of mpox research. Navigating varying regulatory requirements, lengthy review processes and administrative bottlenecks has created setbacks, affecting data collection, intervention strategies and overall response efforts. These delays not only slow scientific progress but also reduce the impact of research in informing public health decisions. Establishing streamlined, harmonised ethical review processes across countries, fostering collaboration between ethics committees and implementing expedited review mechanisms for urgent public health research can help mitigate these challenges.

Surveillance systems for human, animal and environmental health remained largely uncoordinated. Endemic regions face restricted capacity for systematic sampling and testing of potential animal hosts and environmental sources. A lack of standardised protocols for integrated data collection, analysis and sharing impedes a unified understanding of spillover risk and dynamics.

## Future directions and recommendations

To maximise the impact of research, the dissemination of findings through regularly published open-access peer-reviewed journals, reports and regional health conferences to inform policies and interventions is essential. Capacity-building efforts, including continuous training for local researchers and healthcare workers, will improve research methodologies, ethical compliance and data collection practices. Investing in the professional development of researchers not only enhances the quality of studies but also builds a sustainable research ecosystem. These efforts, combined with strategic partnerships and transparent communication, will significantly improve research outcomes and public health responses.

Establishing a designated online platform for the regular upload of research status will promote transparency, facilitate real-time knowledge sharing and improve coordination among stakeholders. Additionally, creating mechanisms to regularly track research progress will help identify challenges early and ensure timely interventions. Sustained community engagement before, during and after research activities is crucial for building trust, ensuring participation and facilitating the effective implementation of studies. Educating local communities about the importance of research helps dispel misconceptions and fears, fostering greater acceptance of public health interventions. Open dialogue with communities can also provide valuable insights into local perceptions, guiding researchers in designing culturally sensitive and impactful studies. Strengthening collaborations with regional and international organisations enhances resource sharing, expertise exchange and overall research efficiency, ensuring a more coordinated response to outbreaks.

Beyond the operational challenges identified, sustained investments in human resource development and laboratory systems remain essential for strengthening outbreak research capacity in Africa. Expanding opportunities for research training, mentorship and workforce development, alongside improving laboratory infrastructure and diagnostic capabilities, will enhance the ability of Member States to rapidly initiate and conduct high-quality research during public health emergencies.

## Conclusion

Research, integrated into outbreak responses, plays a crucial role in rapidly generating evidence and supporting response efforts. The mpox research demonstrates the vitality of collaborative research efforts, the need for continuous systematic monitoring of its progress, and the need for real-time dissemination of research findings to inform practices that ensure effective response to mpox. However, ethical approval of research projects must be fast-tracked, particularly during emergencies. Funding for research is also equally important as funding for disease prevention and control efforts. Real-time dissemination of research findings, starting with early community engagement, is critical to ensure research findings are translated into actions.
